# Evaluation of surface roughness and nano hardness in root dentin on exposure to different irrigating agents - An *in vitro* study

**DOI:** 10.6026/973206300214564

**Published:** 2025-12-15

**Authors:** Dhana Prakash Gurram, Sri Hari Devalla, Ravi Kumar Janga, Vankala Gautam, Saveri Katragadda, N. Ratna Kamal

**Affiliations:** 1 Department of Conservative Dentistry and Endodontics, Drs Sudha and Nageshwara Rao Siddhartha institute of Dental Sciences, Gannavaram, Andra Pradesh, India; 2 Department of conservative dentistry and Endodontics, St Joseph dental college, and hospital, Eluru, Andra Pradesh, India; 3 Department of Orthodontics and Dentofacial Orthopaedics, Drs Sudha and Nageshwara Rao Siddhartha institute of Dental Sciences, Andra Pradesh, India

**Keywords:** Chloroquick, modulus of elasticity, nano hardness, surface roughness

## Abstract

Root dentin integrity can be altered by various endodontic irrigants, potentially influencing long-term treatment outcomes. Therefore, it sis of interest to evaluate the effects of different irrigating solutions on the surface roughness and nano hardness of root dentin in 60 extracted maxillary central incisors. Specimens were treated with normal saline, sodium hypochlorite, EDTA + NaOCl, or Chloroquick, followed by surface and nano-mechanical analysis. Chloroquick demonstrated the highest surface roughness, nano hardness, and modulus of elasticity, followed by EDTA+NaOCl, NaOCl, and saline. Thus, Chloroquick caused the greatest dentin alterations compared with other irrigants.

## Background:

A lucrative endodontic treatment requires proper instrumentation, removal of infected tissues, and three-dimensional obturation of the canal system, paired with appropriate irrigating agents, to ensure successful root canal therapy and elimination of apical periodontitis. Irrigation is an indispensable step to achieve effective disinfection. Chemo-mechanical debridement of the root canal system is facilitated by instruments and efficient irrigating solutions, aiming to produce a clean, debris-free canal suitable for subsequent obturation [1]. Shaping and cleaning of the canal is a decisive phase, as the preparation geometry directly influences the efficacy of all subsequent procedures. Mechanical shaping provides space for medicament delivery, removes infected dentin, and creates an optimal canal configuration for obturation [2]. However, studies conclusively demonstrate that mechanical instrumentation alone-even with advanced Nickel-Titanium systems-cannot adequately disinfect the entire canal system [3]. Therefore, irrigants play a pivotal role in eliminating microbiota. Over time, multiple irrigating chemicals have been proposed, yet the ideal irrigant should kill bacteria, dissolve necrotic tissue, lubricate the canal, remove the smear layer, and remain biocompatible with periapical tissues [4]. Irrigants offer several benefits: removal of particulate debris, lubrication of canal walls, microbial destruction, Dissolution of organic matter, smear layer removal exposing dentinal tubules, and improved cleaning of areas inaccessible to instrumentation. Although irrigants may readily kill microorganisms in the pulp chamber or coronal canal during early preparation, bacteria in deeper or anatomically complex areas may persist and continue to cause apical periodontitis. Their eradication is dependent on both canal shaping and the efficacious delivery of irrigants [5]. Traditionally, irrigation has been performed using passive syringe-and-needle delivery. However, passively delivered solutions advance only about 1 mm beyond the needle tip [6]. Enlarged apical preparations allow deeper needle penetration, improving debridement, but the apical third-especially in narrow or curved canals-remains challenging to clean thoroughly [7, 8, 9]. Kara Tuncer A and Tuncer S, reported that irrigants penetrate only up to 300 µm into dentinal tubules [10], while bacteria may infiltrate tubules up to depths of 1000 µm [11]. Ultrasonics have expanded their utility in endodontics, from refining access cavities to locating canal orifices and activating irrigants [12]. Passive ultrasonic irrigation (PUI) involves filling the chamber and canals with irrigant, then placing an ultrasonic tip to generate acoustic streaming, transient cavitation, and microstreaming-all enhancing irrigant penetration and cleaning efficacy [13]. Chloroquick is an innovative one-step irrigant based on the concept of continuous chelation. It combines stabilized sodium hypochlorite, a buffering system, 1-hydroxyethane-1,1-diphosphonic acid (HEDP), detergent, and system activators. Provided as a two-bottle formulation, it is mixed according to manufacturer instructions. Irrigants can alter dentin surface roughness, which influences dentin wettability and material bonding characteristics [14]. Additionally, dentin hardness has an inverse relationship with tubule density-reduced hardness weakens elasticity and flexural strength [15]. Therefore, it is of interest to evaluate the changes in dentin microstructure following exposure to different irrigating agents.

## Materials and Methods:

Ethical Clearance was obtained from the institutional ethical committee with reference number CEC/05/2018-19. Sixty maxillary central incisor teeth, extracted for periodontal reasons were collected and stored in 0.1% Thymol solution for disinfection for 2 hours and then in normal saline. After clinical and radiological examination, fully formed maxillary central incisors which are Intact, non-carious, no cracks and defects and radiographically patent canals were included. Grossly destructed teeth due to caries or non-carious lesions and radiologically calcified canals and root resorptions were excluded. Armamentarium used are Airotor handpiece (NSK 506c, Japan), Round bur (SS white burs), Endodontic explorer DG-16, Gates glidden drills #2, #3, #4 and #10 - #50 k files (Mani Inc., Japan), Side vented irrigation needle (NaviTip), Ultrasonic handpiece and tips, Diamond disc, Measuring scale, BP Blade no. 15, and handle. Materials used in the study are 5.25 % Sodium hypochlorite, 17% Ethylenediamine tetra acetic acid, 0.9% Normal Saline, Chloroquick HIGH (Neelkanth Dental and surgical Factory, Jodhpur, India), 0.1% thymol solution, cold cure resin, Hydrophilic vinyl polysiloxane impression material.

## Specimen mounting:

All roots were inspected under magnification to ensure that are free from root caries, hypercementosis, open apices, fractures, or craze lines. The root specimens of central incisors were embedded in hard wax and then were inserted into the block of wax to simulate periodontal ligament.

## Shaping and cleaning of the root canals:

For all the 60 samples, after access opening, canal patency was checked by using endodontic explorer DG-16. To standardize the working length, a size 15 k file was inserted into the canal until the tip was first visualized at the apical foramen. Working length was considered 1 mm short of file length. Shaping was done using the crown down technique. The coronal 2/3rd of root was shaped using gates glidden drills sequentially in decreasing order, then apical preparation was done with stainless steel K files. The canal was carefully scouted and fully negotiated to its radiographic terminus with sizes #15 and #20 K files, then the apical one-third preparation was done up to size #50 stainless steel K file of 2% taper. Irrigation was performed witha side vented needle of 27 gauge.

## Grouping of the samples:

Based on the irrigation protocol used, all the 60 samples were divided into 4 groups containing 15 specimens in each group (n= 15).

Group 1: 0.9% Normal saline.

Group 2: 5.25% Sodium hypochlorite

Group 3: 5.25% Sodium hypochlorite with 17% EDTA

Group 4: Chloroquick(high). (1:1 ratio)

Passive ultrasonic irrigation was performed using U -tips sequentially in all the groups.

## Specimen preparation:

The teeth were sectioned longitudinally using a diamond disc under water-cooling with height not more than 20 mm. Thus, 60 specimens were obtained to facilitate the Nano hardness and another half for analyzing the surface roughness. The specimens were embedded on the resin with a prepared surface facing outside.

## Surface roughness testing:

The surface roughness was analyzed using surface profilometry(SURFTEST SJ-201P).The sample were placed on the flat table surface, and the needle of roughness tester was placed on the mid root region of the tooth and surface roughness values of root dentin was recorded. These values were expressed as Ra (µm). The Ra parameter describes the overall roughness of the surface and can be defined as the average arithmetical value of all absolute distances of the roughness profile from the center line within the measuring length.

## Nano hardness testing:

The Nano hardness of each specimen was measured using a Nano indenter, G200; KLA Tencor (CSM Instruments, Switzerland) with a Berkovich diamond tip (TB15269) at a loading rate of 80 mN/min. The indenter was progressively pressed over the sample up to a maximum of 160 mN. Each specimen had five indentations, at an average depth of 3 µm. Nanohardness values were automatically calculated using CSM Instruments Indentation Software (Version 5.14) and reported in MPa. The measurement of the nanohardness values was repeated for each specimen using the same protocol.

## Statistical analysis:

Statistical analysis was performed using the SPSS version 20.0 software package (SPSS Chicago, IL, USA). Confidence intervals were set at 95%, and P values of < 0.05 were interpreted as statistically significant.

## Results:

One-way ANOVA analysis was used to compare four different irrigating solutions on the surface roughness and nano hardness of the Root canal dentin. Tukey's post hoc test was performed to find the statistical significance (Table 1 - see PDF). Intergroup comparison of surface roughness showed statistically significant difference between the groups. Group 4 *i.e.* Chloroquick has shown maximum surface roughness followed by group 3 *i.e.* 5.25% NAOCL + 18% EDTA, group 1 *i.e.* Normal saline, group 2 *i.e.* 5.25% NAOCL and groups respectively (Table 2 - see PDF). Group1: 0.9% normal saline, Group 2: 5.25% NaOCl, Group 3: 5.25% NaOCl+ 17% EDTA, Group 4: Chloroquick(HIGH). Inter group analysis reveals that there is statistically significant difference between the groups in terms of nano hardness. Group 4 (chloro quick) has shown maximum Nano hardness followed by group 2 *i.e.* 5.25% NAOCL, group 1 *i.e.* Normal saline and group 3 *i.e.* 5.25% NAOCL + 18% EDTA groups respectively (Table 3 - see PDF, 4 - see PDF).

## Discussion:

The knowledge of the mechanical properties of dentin are vital because to analyze where occlusal forces are distributed through the tooth and to predict how the absorption of these stresses is modified by age, pathological processes, and therapeutic procedures. Factors altering of surface roughness of root dentin are shaping instruments and techniques, and the irrigating solutions used. Root canal shaping causes potential changes in the root dentin surface due to mechanical load created during instrumentation. In endodontic treatment, rough surfaces could be a clinical benefit in micromechanical bonding of the adhesive materials that need the irregularities on the surface [27, 14]. However, excess surface roughness might cause the adhesion of bacteria leading to plaque formation [28]. The importance of the surface roughness is, to study the relation between the surface geomorphology and wettability of the dentin [14]. Specific treatment procedures like irrigations can affect the structural and mechanical properties of the dentin. Teeth have a structure that ranges from micro to nanometer scale. The nanoindentation method helps in probing and relating the structural and mechanical properties of teeth, so probing of the tooth by the nano indenter helps in determining the modulus of elasticity and hardness at the sub-micron level. Nano hardness determination can help in providing evidence of mineral loss or gain in dental hard tissues as it depends on the amount of calcified matrix per square meter. Reduction in nano hardness can lead to a decrease in fracture resistance [15]. In the present study, Maxillary anterior teeth were selected, as they have adequate remaining dentin thickness after root canal shaping and to overcome the drawbacks like ledge formation, transportation, and perforations. All the extracted teeth were placed in 0.1% Thymol solution for disinfection for two hours and then stored in normal saline at room temperature. The teeth were mounted in acrylic blocks with polyether impression material to simulate bone and periodontal ligament so that there will be no cracks or fractures on the root dentin. The periodontal ligament was simulated to prevent the stress concentration and transfer the functional stresses evenly on the root dentin. The bone was simulated by the acrylic block to reduce the stresses caused by bending movements, which helps in the absorption of the load. The Crown down approach was recommended to reduce the torsional load on the root dentin. Several irrigating solutions like normal saline, EDTA, NaOCl, Citric acid, maleic acid, etidronic acid, chlorhexidine gluconate, hydrogen peroxide, etc., were used traditionally. Unnikrishnan *et al.* 2019 stated that the irrigants react with hydroxyapatite crystals of root dentin leading to changes in micro and nanomechanical properties and ca/p ratio of root dentin [16]. Ballal *et al.* 2019 stated that a decrease in mineral content reduces the microhardness but increases the permeability and solubility of root dentin. So microhardness can provide evidence of loss or gain in the mineral content [17]. Chloroquick is a newer irrigating solution that contains 5.25% NaOCl and 18% HEDP and activator. According to the manufacturer's instructions, Chloroquick helps in the removal of the smear layer and the eradication of microbes. NaOCl has tissue dissolving and antimicrobial properties, and EDTA is a chelating agent that helps in smear layer removal, whereas Chloroquick has both the properties of tissue dissolving and smear layer removal. Hence Chloroquick is used as an irrigating solution in the present study. The different irrigating agents used in this study are 0.9% normal saline, 5.25% NaOCl, 17% EDTA, Chloroquick (HIGH), which were used to analyze the geometric alteration, nano hardness, and the modulus of elasticity of the root dentin. Cruz-Filho *et al.* 2002 stated that 0.9% normal saline was used as a control because it does not cause any chemical changes on the dentin. In the present study, all the irrigants were used for five minutes [18]. Ulusoy & Gorgul, stating that this duration is like the clinical scenario [19]. Tatari *et al.* 2013, concluded NaOCl treatment did not modify the roughness of the dentin surface when used before or after the chelating agents, consistent with previous findings that NaOCl does not cause decalcification [20]. The sectioning method was most widely used to evaluate the root dentin morphology. The advantage of the sectioning method is sample preparation at a very low expense and less time consumption. The disadvantages are the formation of cracks, craze lines, and loss of area of interest. The teeth were separated longitudinally using a diamond disc underwater coolant. Water coolant was used to eliminate the charring of the root dentin. Several authors stated that irrigation with NaOCl and in the combination of NaOCl and EDTA significantly increases the roughness of dentin and it changes the surface properties of root and coronal dentin, such as wettability, which influence the adhesion of bacteria. So wettability is strongly dependent on surface roughness [14, 21 and 22]. In the present study, with respect to surface roughness one way ANOVA analysis showed statistical significance between the groups (Table 3 - see PDF). There is a slight increase in surface roughness with these chemical solutions leading to the structural change of root dentin. NaOCl has shown a slight increase in the surface roughness value. The increased surface roughness is due to the removal of collagen similar to that of the etched enamel, and it increases the wettability of dentin, which is a dependent factor of roughness. Attal *et al.* stated that the increase in wettability after NaOCl treatment could be explained because deproteination leads to a hydrophilic surface [23]. The surface roughness values of NaOCl and EDTA (group: 3) used in combination have shown similar results when compared with Chloroquick group (group: 4) and slightly increased when compared with NaOCl (group: 2) and saline groups (group: 1). This explains increased surface roughness values in group 3 are because of EDTA, which helps in the dissolution of the inorganic component, so the dentinal tubules become patent due to the removal of smear layer [24]. Chloroquick (HIGH) has shown increased surface roughness when compared to other groups due to HEBP, which acts as a chelating agent, and NaOCl, which is an antimicrobial agent that helps in dissolving the remnant tissues [25]. Dentinal surfaces, including root canal walls of all the samples tested for nano hardness, were polished prior to nanohardness testing, similar to sample preparation methods of previous studies. However, polishing reduces the thickness of dentinal surfaces and may influence the values of nano hardness, but the teeth selected have similar dimensions, the thickness of dentinal blocks was standardized. Russel *et al.* 2014, suggested that the hardness of root dentin depends on the density of dentinal tubules and the amount of intertubular and peritubular dentin [26]. The accomplishment of root canal therapy is contingent on instrumentation, irrigation, disinfection, and three-dimensional obturation of the root canal. For removing the pulp tissue remnants and dentinal debris, one should use instrumentation and irrigation simultaneously. The main objective of irrigation is to reduce the smear layer. The smear layer cannot be reduced adequately using a single irrigating solution. Thus a combination of irrigants used in succession, simulating the clinical conditions. As irrigating solutions influence the physical and chemical properties of root dentin like hardness, roughness, permeability, and wettability. The advanced concepts of chemo mechanical preparation imply that chemical irrigants should be applied on instrumented root canal surfaces to remove the smear layer. NaOCl is not a chelating agent, but it can also modify the calcium/phosphate ratio in radicular dentin. NaOCl is a traditional irrigating solution in endodontic treatment. It can eliminate organic residue in the smear layer and small and narrow canals that cannot be accessed by biomechanical instrumentation. Ethylenediamine Tetra Acetic Acid (EDTA) is a chelating substance that helps to remove calcium ions of the dentin, leading to demineralization so that it increases the dentin permeability of the root canals. Chloroquick (HIGH) is a new formulation of 5.25% sodium hypochlorite with 18% hydroxy ethylidene bisphosphonate (HEBP) is a newly formulated irrigant. It is available in 2 vial system, which is then mixed according to the manufacturer's instructions. In the present study Chloroquick (HIGH) has been used because of better removal of smear layer when compared with conventional irrigation protocol [27]. Cheron *et al.* stated that the nano-indentation test allows measurement of the modulus of elasticity and hardness of the small media and can characterize early damage to the hard tissues caused by external factors, so nano indenter helps in providing more accurate information on smaller areas of dentinal surface and it can easily assess the intertubular dentin [1]. Tissue dissolving effect of NaOCl (group: 2) was protected by hydroxyapatite, but in group 3, *i.e.*, NaOCl with EDTA, collagen will be exposed to chelation property of EDTA, and tissue dissolving capacity of collagen fibers increase the reduction of nanohardness and increases the erosion. Giardino *et al.* 2015 stated that demineralizing effect of irrigants mainly depends on surface tension determining the wettability. Wettability is the ability of fluid to spread over, and it is an important factor for the penetration of irrigant into dentinal tubules [28]. Saghari *et al.* 2009 reported that penetration depth determines the hardness reduction [29]. In the present study, Chloroquick (HIGH), that is group: 4, when compared to other groups (group: 1, 2 and 3) shown an increase in the nano hardness, mainly due to HEBP, which has high wettability and high penetration into dentinal tubules. Thus, we hypothesize that the complete removal of the smear layer increased the sealing effect and the bond strength of the resin-based root canal sealers to dentine, which can be compensated for the reduction or loss in the mechanical properties after using these irrigation protocols.

## Limitations of the study:

It was demonstrated that the biomechanical response of root dentin to root canal instrumentation was influenced by dentin dehydration, where they found no residual micro strain concentrations in hydrated roots after canal instrumentation [30]. Recently American association of tissue bank recommended a storage temperature of -200 C for up to 6 months of storage and -400 C for longer periods of deep-frozen preparation [31]. These storage temperatures were not maintained in this study. The section of the samples was done using diamond disc under water coolant, hence the roughness of the disc also may alter the surface roughness values of the root dentin surface.

## Conclusion:

Within the limitations of the present study, it can be concluded that Chloroquick (HIGH) has shown more surface roughness and nano hardness, when compared with 0.9% normal saline,5.25% sodium hypochlorite, 5.25% sodium hypochlorite and 17% Ethylenediamine Tetra Acetic Acid.
